# Handbook of Obstetric Nursing

**Published:** 1890-06

**Authors:** 


*Handbook of Obstetric Nursing.* By FRANCIS W. N. HAULTAIN, M.D., and J. HAIG FERGUSON, M.B. Edinburgh: YOUNG J. PENTLAND. 1889.

It seems that this work is not intended so much for the monthly nurse with whom in this neighbourhood we are acquainted, as for the midwife who has the sole charge of an ordinary case of labour.

But for either of these persons the book contains more than is necessary, and so presents an element of danger. It is not that we take much exception to any part of it as a dissertation on the subjects treated; but the midwife who has a trained doctor to whom to appeal will be no better able to go through her work because she has a smattering of knowledge about the development of the decidua, or the various measurements of the fœtal head; and the monthly nurse, learning her business from a book of this kind, yet having to carry out the instructions of various medical attendants, is very likely to produce unpleasant worry by putting the lecturer's particular views about unimportant details against the practice of each doctor whom she meets.

The observations about compression of the uterus in the third stage of labour require some modification after the investigations recorded in the second volume of the Laboratory Reports of the Edinburgh College of Physicians. With this exception, the book under review would form an excellent students' introduction to the practice of midwifery.

We always look for some amusement in a glossary; but here it is almost limited to the definition of bladder, which is described as "The receptacle for the urine,"—a definition which would serve for a breakable utensil provided by art and supplied by ordinary commerce.

